# Dinosaur skull geometry does not follow functional optimization trends but facilitates adaptability

**DOI:** 10.1016/j.isci.2025.114115

**Published:** 2025-11-19

**Authors:** Stephan Lautenschlager, Minnie M. Cione, Grace Cowley, Benjamin J. Creasy, Harrison Everard, Oliver J. Graves, Poppy E. Hawkins, Alisa Plaksina, Helena N. Rowlstone, Austin M. Smith, Lauren M. Steward

**Affiliations:** 1 School of Geography, Earth & Environmental Sciences, University of Birmingham, Birmingham, UK; 2 The Lapworth Museum of Geology, Birmingham, UK

**Keywords:** natural sciences, paleontology, evolutionary biology, paleobiology

## Abstract

Dinosaurs exhibited a remarkable array of skull shapes and sizes. However, the geometry of the cranial skeleton and its different functional units (rostrum, orbital region, and braincase) has not been quantified biomechanically. Furthermore, it is unclear if skull evolution followed a trend toward optimization of feeding performance and structural integrity. Here, we used morphometric analyses, theoretical morphospaces, and biomechanical modeling to quantify skull geometry and investigate how skull proportions shaped dinosaur evolution. Our results demonstrate that skull proportions followed a narrowly constrained pattern, with the rostrum showing the largest variability and plasticity. Dinosaur skulls did not evolve toward functional optimality but evolved within a “Goldilocks” zone that represented a compromise of different functions. Broad adaptability of the rostrum may have been beneficial during the transition from non-avian dinosaurs to birds, with the rostrum serving as a focal point for environmental manipulation due to the loss of forelimb function for flight.

## Highlights

• Dinosaur had variable skull proportions and dimensions
• The snout (rostrum) was highly variable and adaptable
• Dinosaur skulls did not evolve theoretically optimal skull proportions
• The adaptability of the rostrum may have been beneficial during bird evolution

## Graphical abstract

**Figure:**
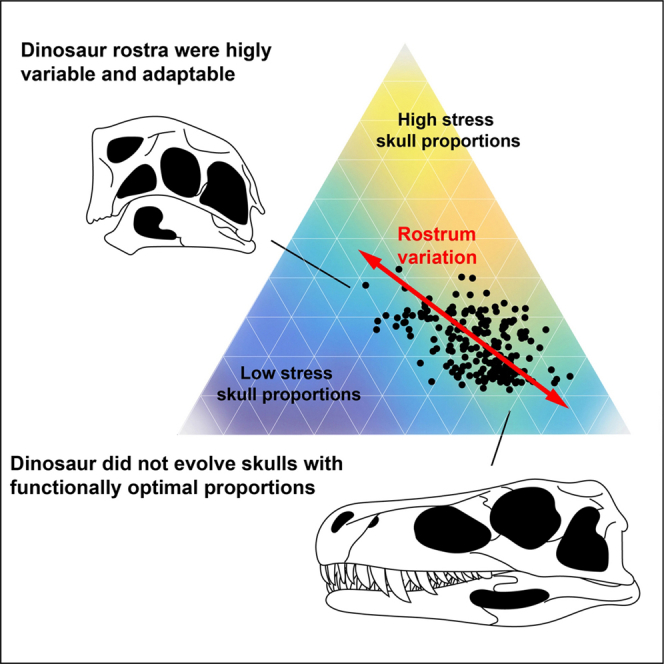

## Introduction

Throughout their 165-million-year reign, dinosaurs achieved a remarkable range of morphological diversity.^1^^,^^2^^,^^3^^,^^4^^,^^5^ While this diversity is most apparent in body size variation, it is also expressed in the range of shapes and sizes of the cranial skeleton.^6^^,^^7^^,^^8^ Variability in skull morphology has been linked to dietary adaptations, feeding strategies, and niche partitioning.^9^^,^^10^^,^^11^^,^^12^ Consequently, previous studies have investigated and quantified the morphological variation of dinosaurian and related archosaurian/archosauromorph skulls.^13^^,^^14^^,^^15^^,^^16^^,^^17^ However, these studies primarily focused on overall skull shape and/or size, whereas the geometry and dimensions of the functional units (rostrum, orbital region, braincase) making up the cranial skeleton have received very little attention in comparison. Marugán-Lobón and Buscaloni^18^ quantified the skull geometry of different extant and fossil archosaur species but included only a small number of dinosaur species among the sample. Other studies have focused on individual cranial components, such as the orbit^19^^,^^20^ but have done so in isolation and not in relation to other skull regions. As a result, broad patterns in the evolution of dinosaur skull dimensions and proportions remain unquantified, leaving several key questions unresolved. These include whether (1) skull geometry is driven by ecological and/or functional factors, and (2) the variation in skull proportions is constrained by phylogeny. Furthermore, the functional implications of different skull dimensions and ratios of the individual components have not been tested systematically in a biomechanical context. This study quantifies dinosaurian skull geometry using a sample of 204 species and evaluates functional properties such as cranial stability, bite force, and mechanical advantage through finite element analysis (FEA). Theoretical models are used to compare empirical and realized skull morphologies and to assess whether skull evolution is directed toward functional optimality; defined as the minimization of cranial stresses in the context of comparative feeding scenarios. Integrating morphological and biomechanical results within a performance space framework allows us to identify how changes in skull dimensions and proportions impact feeding performance and possible selection pressures.

## Results

### Skull proportions

**Figure 1:**
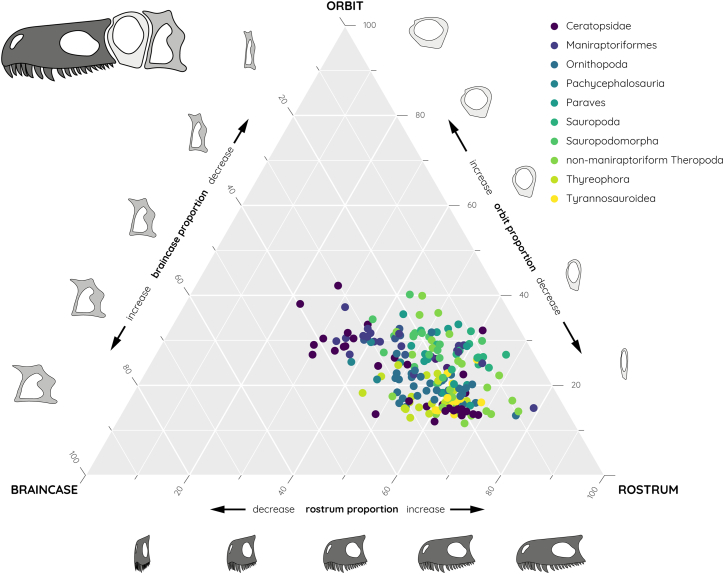
Empirical ternary morphospace distribution of different dinosaur species along variable skull proportions

The analysis of over 200 dinosaur species across different groups demonstrates that the proportions of the tested cranial regions vary only along a narrow trajectory. Of the overall ternary morphospace, dinosaurs only occupy a region representative of 19% (Figure 1). The greatest variation is found in the length of the rostrum, ranging from a minimum value of 22% (of the skull length) to a maximum of 78% (Figure 1). In contrast, the orbit (11%–42%) and the braincase (5%–43%) regions are subject to a lower range of variability (Figure 1). Extreme examples for rostral variability include the ceratopsian *Protoceratops* on the lower end of the spectrum and the ornithomimosaur *Deinocheirus* on the higher end. For orbit proportions, the lowest contribution to skull length is found in the allosauroid *Asfaltovenator*, with the ceratopsian *Gobiceratops* at the upper end. Braincase variability is exemplified by *Deinocheirus* with the smallest value and the ceratopsian *Auroraceratops* with the highest value.

**Figure 2:**
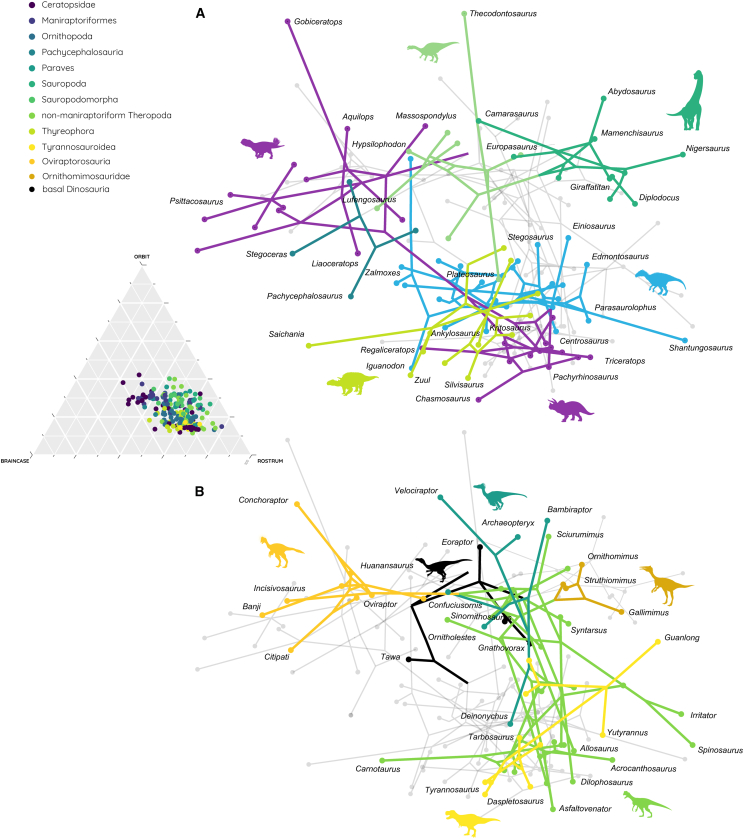
Phylomorphospace distribution based on different skull proportions (A and B) Phylomorphospace shown for (A) ornithischian and sauropodomorph species and (B) theropod species.

Although overall skull geometry is considerably constrained across the dataset, notable differences exist between individual dinosaur groups (Figures 1 and 2). Ceratopsians exhibit the greatest disparity in both relative and absolute terms, with variation across all three skull components. There is a marked shift from basal ceratopsians with short rostra to derived forms with rostra comprising up to approximately 70% of skull length and reaching 800 mm in absolute length (Figures 1 and 2A). This represents an increase in absolute rostrum length of more than 750%. Longirostral morphologies are generally found in theropods (in particular maniraptoriformes and Paraves), some ornithopods and ceratopsians, and some sauropods. However, in the latter, this is an effect of the rotation and reduction of the orbit and braincase regions rather than an absolute increase in rostral length.

**Figure 3:**
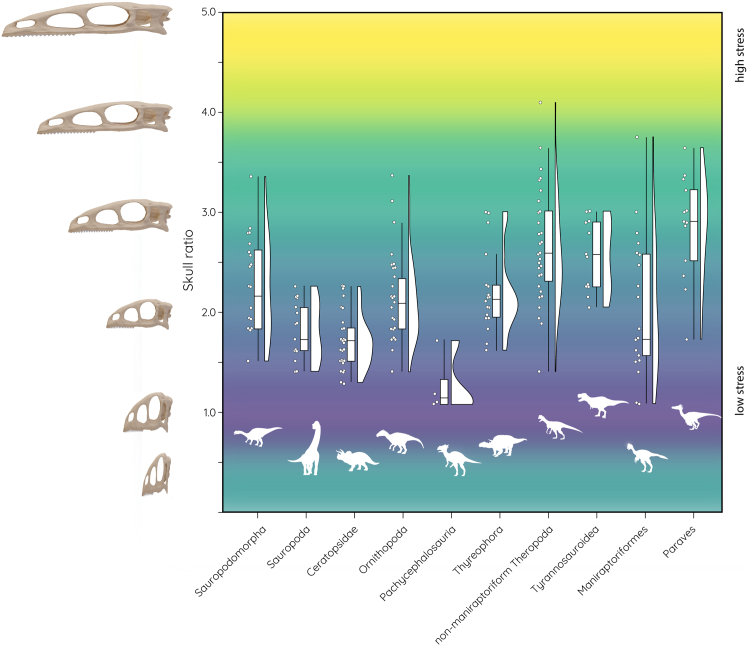
Skull ratio (length-to-height) distribution across different dinosaur groups. Theoretical skull models used in the biomechanical analyses depicted alongside the *y* axis

In contrast, the length-to-height ratios of all studied species show considerable variation and range from 1.1 to 4.1 but with a cluster around the mean of 2.1 (Figure 3). Distinct trends and differences are visible between the studied dinosaur groups. Notably, pachycephalosaurs, ceratopsians, and to some extent sauropods have largely uniform skull ratios with values between 1.0 and 1.5 (Figure 3). In comparison, sauropodomorphs, ornithopods, and particularly theropods (both basal and derived) have elongated skulls with ratios over 2.0 (Figure 3).

**Figure 4:**
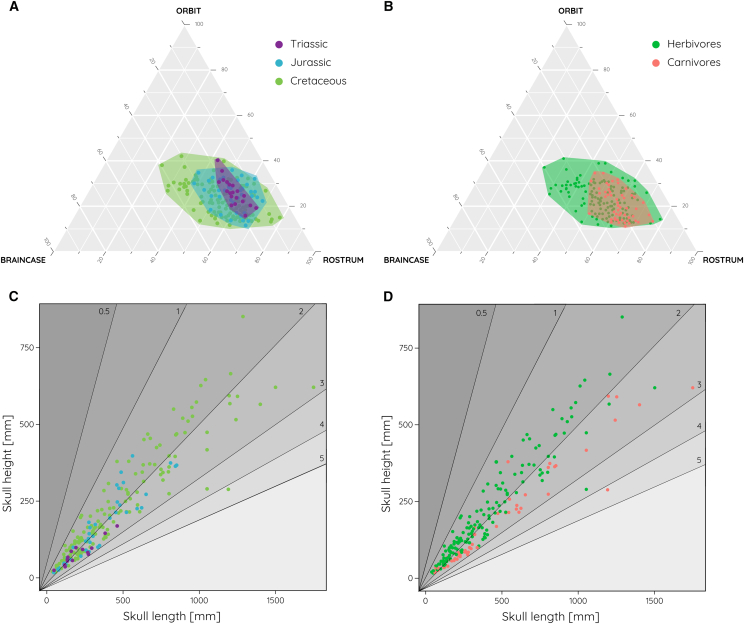
Skull proportions in the context of geological time and diet (A–D) (A and B) Ternary morphospace and (C and D) skull ratio distributions shown in a (A and C) spatial and (B and D) dietary niche context.

Although there is no clear association between skull proportions and geological age, there is an expansion of the morphospace during the Jurassic and particularly in the Cretaceous. Interestingly, this expansion is driven by the relative changes in orbit and braincase regions rather than rostral dimensions (Figure 4A). However, this observation may be biased by lower sampling of Triassic species. Similarly, herbivorous species occupy a larger portion of the morphospace than carnivores (Figure 4B).

When looking at skull ratios, Triassic species are restricted to values around 2.0–2.5. It is not until the Jurassic and more prominently during the Cretaceous when different skull ratios toward the lower (1.0) and higher end (3.5) were explored (Figure 4C). In terms of diet, there is a subtle trend for herbivorous species to have shorter skulls, whereas carnivores evolved more elongated skulls (Figure 4D). The separation is around a value of 2.0–2.5 but with considerable overlap between the two groups.

### Functional analyses

**Figure 5:**
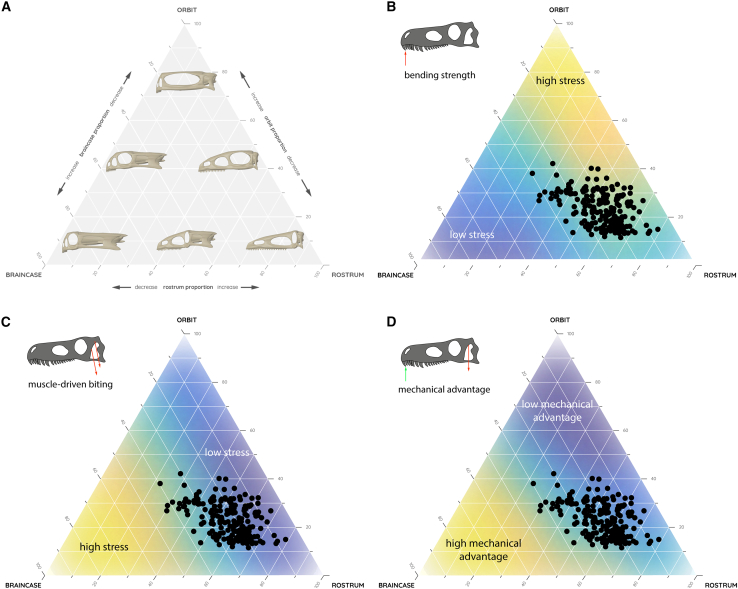
Functional morphospace distributions (A) Theoretical ternary morphospace distribution with selected hypothetical models mapped onto corresponding skull proportions. (B–D) Biomechanical performance morphospaces based on the results of the finite element analyses with species distribution mapped for (B) bending strength, (C) muscle-driven biting, and (D) mechanical advantage.

The functional performance in the context of different feeding and biting scenarios was tested for 34 hypothetical skull models that represent the entirety of the ternary morphospace plot (within a resolution of 10% variation of skull proportions). Skull bending and muscle-driven biting scenarios were tested for all models and average von Mizes stress and mechanical advantage were measured as performance indicators and a proxy for skull stability. Results from both biomechanical analyses demonstrate that brevirostral models with short orbital but expanded braincase regions experienced the lowest bending stresses whereas brevi-to mesorostral models with expanded orbital regions recorded the highest stresses (Figure 5A). For muscle-driven biting, this situation is reversed and stress susceptibility correlated strongly with braincase length, with expanded braincase models experiencing the highest stresses (Figure 5B); the same was found for mechanical advantage (Figure 5C). When compared to the actual species, their distribution in morphospace was found to consistently occupy a region of moderate stresses and mechanical advantage between the extreme values for all tested scenarios. None of the species in the dataset occupies regions of extreme stresses, which are dominated by theoretical morphologies with extensive orbit and/or braincase dimensions. The relation between stress susceptibility and high mechanical advantage (effectively a measure of bite performance) shows that all dinosaur species in the dataset are subject to a trade-off between both measures.

For the different skull ratios, eleven hypothetical models with length-to-height ratios from 0.5 to 5.0 (in 0.5 increments) were tested under the same scenarios. The lowest stress values were found for uniform to slightly elongated skulls with ratios of 1.0–2.0. Values above 3.0 (and also for 0.5) recorded higher/the highest stresses. As for the results for different skull proportions, most species plot along the zone of low to moderate stresses, with only a few species falling within the higher stress regimes.

### Evolutionary and phylogenetic trends

When evaluated in an evolutionary context, the results show that the basalmost dinosaurian taxa plot in the center of the occupied morphospace with rostral proportions around 50% and orbital and braincase dimensions of ca 20%–30% (Figure 2B). However, across the phylogenetic tree, individual groups diversify almost immediately and occupy distinct regions of the morphospace away from the ancestral taxa. Evolutionary movements within these groups are variable, with some showing a quick entry into different morphospace regions. For example, non-ceratopsoid ceratopsians occupy a morphospace toward shorter rostral proportions, whereas the more derived species are found at the opposite end (Figure 2A). Sauropodomorphs show a similar trend with sauropods moving along a decreasing braincase ratio trajectory compared to their ancestral non-sauropod sauropodomorphs. Ornithopods and thyreophorans don’t show any clear trends and largely overlap with derived ceratopsians. Among theropods, non-maniraptoriform species generally occupy the morphospace toward longirostral proportions without clear separation. It is not until the appearance of maniraptoriforms that a distinct occupation pattern emerges. Oviraptorosaurs, ornithomimosaurs, to some extent therizinosaurs (but with only two species having insufficient sample size), and paravians all occupy largely distinct regions along a trajectory of changes of the rostral and braincase dimensions (Figure 2B).

## Discussion

Dinosaur skulls vary greatly in size, shape, cranial ornamentation, exaggerated defensive and/or display structures, and other anatomical features. It could thus be expected that skull proportions and dimensions show an equally high diversity. However, despite the morphological and ecological variability, our analyses demonstrate that both the skull proportions (i.e., relative size of individual cranial regions) and the dimensions (i.e., length-to-height ratio) follow a narrowly constrained pattern. Although skull dimensions vary more widely across groups, this variation is primarily driven by the relative contribution of the rostrum to the overall skull proportions. In this respect, dinosaurs differ substantially from other non-archosaurian vertebrates, such as synapsids, which exhibit greater plasticity in skull configuration, including pronounced rostral shortening and braincase expansion.^21^ This finding is surprising given the large taxonomic diversity of dinosaurs, in which variation and plasticity is focused on the rostrum. Furthermore, it raises the question as to why there is no overall trend toward facial shortening and variation of the orbital and braincase region as in synapsids. The variation of the cranial components in synapsids seems to have been driven by different factors, such as a reorientation of the orbital region more frontally (resulting in fully forward-facing orbits in primates)^22^^,^^23^ and the expansion of the braincase with increasing brain size.^24^^,^^25^ Moving the orbital region forward and aligning it with a shortened rostrum in primates is thought to represent an effective solution to accommodate stresses.^21^^,^^26^^,^^27^ Indeed, the stress distribution of the hypothetical models tested in this study demonstrates that large orbital proportions (>40%) represent a biomechanical liability. In fact, the orbital and braincase regions maintain a proportional balance, with neither exceeding twice the size of the other in dinosaurs and this pattern is also retained in crown-group birds.^21^ Dinosaurs seem to have circumvented the problem of strongly reducing the orbital region (as in primates) by modifying the shape of the orbit itself rather than altering the proportion of the orbital region to withstand high bite forces and resulting stresses.^19^ This may have left the rostrum free to broader morphological changes. On the other hand, substantial expansion of the brain and the braincase did not occur until relatively late in dinosaur evolution, specifically within Paraves.^28^^,^^29^ In comparison, in synapsids, brain expansion occurred already in cynodonts and mammaliaforms but resulted in increased stresses on the braincase region, which required a re-direction of loads to other cranial regions,^30^ a strategy that may not have been feasible in dinosaurs. Furthermore, the skull acts as an important tool in dinosaurs, in particular in the context of forelimb reduction in different theropods.^31^^,^^32^^,^^33^^,^^34^^,^^35^ The lack of manual dexterity in non-avian dinosaurs and the subsequent repurposing of the forelimbs as a flight apparatus retained a focus on the cranial structure, thereby explaining the requirement for rostral plasticity.

The results of the biomechanical analyses using hypothetical models demonstrate that the variation of cranial proportions has different functional consequences. Varying proportions for the orbital and the braincase region each lead to substantial differences in stress susceptibility and mechanical advantage: moving from low stress regimes (small orbital region and large braincase region for skull bending scenarios but large orbital region and small braincase regions for muscle-driven biting scenarios) to high stress regimes (large orbital region and small braincase region for skull bending scenarios but small orbital region and large braincase regions for muscle-driven biting scenarios) in the theoretical morphospace. The converse patterns indicate that it would be biomechanically disadvantageous to have evenly proportioned orbital and braincase regions. In contrast, changes in rostrum length progress along an iso-stress regime for the different tested scenarios, thereby not compromising functional competence and stability. Variation of the rostrum did not result in any of the hypothetical models moving to a low stress regime of the morphospace and thus an optimal condition in the context of the tested scenarios. However, it also did not result in an increase of stresses, keeping the hypothetical skull models in a “Goldilocks” condition, which constitutes a compromise between performances.

This lack of optimization was also noted for theropod dinosaurs by Rayfield and Milner^36^ using stylized models of two different theropods and two crocodilians, finding that most theropod skulls are sub-optimally constructed to resist feeding-induced stresses. However, unlike the mandible, which is predominantly adapted to feeding functions and can be adapted to resist high stresses,^37^ the rostrum represents a compromise between a variety of (competing) functions. The rostrum may, therefore, have to be the most flexible cranial region to allow not only the procurement of food (including hosting teeth or beaks as functional components) but also accommodate the naris (and associated respiratory airways) and the antorbital fenestra.^38^^,^^39^

Assuming ecology played an important role in shaping cranial and, in particular, rostral evolution, it would be expected that species with different dietary adaptations occupied separate parts of the morphospace. Such a morphofunctional separation based on differences in diet and prey preference has been observed in modern canids^40^ with long-snouted species found to be small prey specialists, while short-snouted species are adapted to subdue larger animals. However, similar inferences are not fully supported by our results. Although herbivorous species expand the morphospace compared to their carnivorous counterparts, there is a substantial overlap between both groups. More subtle dietary niche adaptations may have a stronger effect on rostrum shape, but these are difficult to disentangle. Carnivores are generally constrained in adapting the rostrum due to functional requirements of hosting teeth with a predominant slicing and cutting function. Although some variation exists in theropod teeth within this regime (e.g., conical vs. blade-like teeth)^41^^,^^42^^,^^43^ there is little room for deviation from this function. In contrast, herbivores have a broader range of mechanisms to acquire and process plants. This can range from different tooth shapes,^4^^,^^44^ the reduction or complete loss of teeth,^45^^,^^46^ different beak morphologies,^47^^,^^48^ gastric mills,^49^^,^^50^ muscle configurations,^51^^,^^52^^,^^53^ and others. In particular, the requirement for teeth is a constraining factor for adapting rostrum length in theropods, and substantial variation is only found in oviraptorosaurs, which foreshortened the rostrum alongside complete edentulism.^45^^,^^54^ Nevertheless, among tyrannosaurid theropods, there can be some ecological-related differences. Long-snouted alioramines are thought to have been adapted to prey on smaller and more agile prey,^55^ whereas the compact-snouted *Tyrannosaurus* and related large-bodied species were large-prey and bone-cracking specialists.^56^ Similarly, small differences in the relative size of the braincase region and thus the attachment sites of the jaw adductor musculature and correspondingly larger jaw gapes hint at differences in feeding and hunting style in large theropods.^57^

Conversely, variation in cranial dimensions may be controlled and constrained by phylogeny. Brusatte et al.^13^ found in a landmark-based characterization of theropod skull shapes that morphospace distribution was strongly correlated with phylogeny but not biting function (quantified by the mechanical advantage of the mandible). Similarly, maxilla and premaxilla dimensions were found to be not indicative of feeding styles but rather phylogeny in dromaeosaurid dinosaurs.^58^ However, the same study suggested that phylogenetic differences between long-snouted Asian and short-snouted North American taxa may be underpinned by differences in prey selection and/or environmental conditions. Phylogenetic effects may have an impact on the morphospace distribution observed here as well, with several groups distinctly separated by phylogenetic affinities. However, as also noted by Powers et al.,^58^ morphological differences in cranial, and in particular in rostral shape, underpinned by functional adaptations, are commonly used to code taxa in phylogenetic analyses. This makes phylogenetic and functional effects difficult to disentangle, but future studies could investigate these points using more advanced phylogenetic comparative methods.

### Limitations of the study

Of course, skull proportions and ratios alone do not capture the entirety of cranial morphology. Differences within the individual components, such as the size and shape of the various fenestrae,^19^ cranial ornamentation,^59^^,^^60^ tooth shape,^43^ and other features, can be highly variable while maintaining constant skull ratios or proportions. Individual cranial bones may have been adapted to withstand high stresses, such as, for example, fused nasal bones in *Tyrannosaurus*,^61^ without requiring a change in cranial proportions. Other examples include the evolution of narrow and pointed rostra to reduce torsional stresses^36^ and the acquisition of keratinous beaks to mitigate stress magnitudes.^46^ In addition, it is thought that cranial kinesis was present in some theropods, which would act as a further mechanism to reduce stresses.^62^^,^^63^^,^^64^ Considering that many of these adaptations emerged in most maniraptorifoms (with the exception of oviraptorosaurs) and prior to the evolution of Avialae, it may be plausible that the plasticity of the rostral region facilitated the transition from non-avian theropods to birds. In particular, the transformation of grasping hands to wings required a substitute tool for prey and environmental manipulation opening the possibility that the adaptability of the rostrum within functional constraints allowed the vast range of beak morphologies seen in modern birds.^65^^,^^66^^,^^67^

### Conclusions

Dinosaurs evolved a large range of skull shapes and sizes, but our results demonstrate that despite this morphological diversity, the geometry (proportions and dimensions) of the cranial skeleton follows a narrowly constrained pattern. Variation in cranial proportions can primarily be attributed to plasticity in rostrum size (both absolute and relative compared to skull size), whereas the orbital and braincase region show a conservative trajectory. This pattern is underpinned by cranial stability factors as evidenced by the biomechanical performance of hypothetical skull models exploring a range of skull geometries. However, actually realized skull morphologies were found not to evolve toward a functional optimum to reduce feeding induced stresses but evolved within a “Goldilocks” zone that represented a compromise of different functional requirements. These constraints on orbital and braincase proportions and dimensions ultimately channeled the evolutionary changes toward the rostrum. The plasticity and adaptability of the latter thereby influenced not only dinosaur cranial evolution but also may have played a considerable role in the transition from non-avian theropods to birds.

## Resource availability

### Lead contact

Information and requests for resources should be directed to the lead contact, Stephan Lautenschlager (s.lautenschlager@bham.ac.uk).

### Materials availability

Digital models used for the biomechanical analyses have been deposited to a figshare repository and are available at figshare (https://doi.org/10.6084/m9.figshare.30415987).

### Data and code availability

• Data: All data and measurements are available in Table S1.
• Code: This paper does not report original code.
• Other: This paper does not report any additional resources.

## Acknowledgments

We would like to thank Andre Rowe and two anonymous reviewers, as well as the editor, who provided very helpful and constructive feedback on previous versions of this manuscript.

## Author contributions

S.L. and M.M.C. conceived the project. All authors performed data collection of skull dimensions and proportions. S.L. performed the biomechanical analyses. S.L. wrote the first draft of the manuscript. All authors approved the manuscript for publication.

## Declaration of interests

The authors declare no competing interests.

## STAR★Methods

### Key resources table

**Table:** 

REAGENT or RESOURCE	SOURCE	IDENTIFIER
**Deposited data**		

List of sampled species and measurements	This paper	Table S1
Digital models	figshare https://figshare.com/	https://doi.org/10.6084/m9.figshare.30415987

**Software**		

R (v.4.3.0)	R Core Team	https://www.r-project.org/
RStudio (v.2023.12.1.402)	Posit Team	https://posit.co/download/rstudio-desktop/
Blender (v. 4.3)	Blender Foundation	www.blender.org
Hypermesh (v.11)	Altair	https://altair.com/
Abaqus (v.6.141)	Simulia	https://discover.3ds.com

### Experimental model and study participant details

#### Morphometric dataset

The following measurements were taken to quantify dinosaurian skull geometries: (i) skull length, defined as the horizontal distance between the premaxillary symphysis (=tip of the skull) and the posteriormost extent of the quadrate bone, (ii) rostrum length, defined as the horizontal distance between the premaxilla and the center of the lacrimal bone, demarcating the boundary to the orbit; (iii) orbital region length, defined as the horizontal distance between the center of the lacrimal and the center of the postorbital/jugal contact; (iv) brain case length, defined as the horizontal distance between the postorbital/jugal contact and the posteriormost contact between the quadrate and the squamosal; (v) skull height, defined as the vertical distance between the base of the skull formed by the maxilla/jugal margin and the dorsal extent of the frontal/parietal contact. For species with extended ornamentation (Ceratopsia, Hadrosauridae, some Theropoda), the exaggerated structures were excluded from the measurements. In addition, the temporal, dietary, and taxonomic affinities for each species were collected.

All measurements were taken from relevant literature sources depicting the skull of each species in lateral view. Overall, data were collected for 204 species. All data were collected by ten undergraduate students as part of a course-based research project embedded into a year 1 module in a Palaeontology and Geology program over a period of eleven weeks.^68^ For this task, each student was assigned a group of approximately 30 species. The species in each group were distributed differently for each student, and each species was assigned at least twice to allow for redundancy and to identify outliers in the data collection. Training on data collection, measurements, and the identification of anatomical structures was provided by the senior author and the progress, and accuracy of the data collection were regularly checked. Additional data were collected by the senior author to fill in gaps in the dataset.

### Method details

#### Biomechanical models

In order to test the effect of different skull dimensions and varying proportions of the three skull units of interest, different digital models were constructed. For the base model, a skull shape modeled after the dinosauriform *Asilisaurus kongwe* was selected.^69^ This species was chosen as it represents one of the candidates for a last common ancestor of Dinosauria^70^ while also showing a skull morphology free of obvious ornamentation, exaggerated structures, and other ostentatious adaptations. Furthermore, *Asilisaurus* presents a stage in dinosaur evolution pre-dating extensive diversification and may therefore represent the base Bauplan from which different modifications arose. It is therefore ideally suited for testing the functional effects of different skull dimensions and how these may have influenced subsequent cranial evolution.

The base model was constructed using a box-modelling approach^71^ in Blender 4.3 (www.blender.org). The rostrum, orbital region, and braincase were subsequently separated into individual components using a Boolean modifier and follow the same subdivision as for the morphometric measurements outlined above. The base model has a ratio of 60/20/20 (rostrum/orbit/braincase). In the next step, the dimensions of each component were successively changed in 10% steps. An increase of one component by 10% required the decrease of one of the other two components. This variation was modeled to represent all possible permutations for 10%-step changes. A size of any component below 10% was deemed impossible and was therefore not modeled. Overall, this resulted in 34 hypothetical models.

The constructed models were subjected to biomechanical simulations using finite element analysis (FEA). All models were exported as STL files and imported into Hypermesh 11 (Altair) for solid-meshing and the setting of boundary conditions. Each model had an element count of ca. 300,000 tetrahedral elements. All models were constrained from movement at the quadrates at four nodes on each side. Material properties were selected to represent crocodilian bone (E = 15.00 GPa, ʋ = 0.29).^72^ Two functional scenarios were tested: (i) a simplified bending test with a load of 100 N applied to the tip of the snout in dorsal (z) direction. While the magnitude of the load force is not important in a purely comparative context, it was selected to fall within the range of bite force estimates for a species of this size (e.g., comparable to the basal saurischian *Eoraptor lunensis*).^73^ The load was adjusted for each model to account for differences in skull surface area following Marcé-Nogué et al.^74^ This scenario represents a change in skull dimensions without a concomitant change in the musculature and tests just the differences in osteological proportions. (ii) To account for the change in muscle mass when changing the skull proportions, a second scenario was simulated. For this scenario, an additional constraint was placed on the first premaxillary teeth symmetrically to record reaction (=bite) forces. Load forces were placed along the margins of the upper temporal fenestra in a simplified simulation of the jaw muscles (specifically the m. adductor mandibulae externus group). Force magnitudes were estimated using a variation of the dry skull method^75^ by measuring the surface area of the adductor chamber in dorsal view. Although this represents a simplification of the more complex actual muscle division, it required no decision regarding the extent of the muscle attachments and their changes with skull proportions, which would lead to increased subjectivity. For the same reason, the pterygoideus muscles were not simulated.

All FEA models were exported as INP files and solved in Abaqus 6.141 (Simulia). Results of the individual analyses were obtained as median von Mizes stress magnitudes and mechanical advantage (=bite force/muscle force) values. These results were subsequently plotted onto ternary diagrams representing the theoretical and actual variations in skull proportions.

### Quantification and statistical analysis

#### Phylomorphospace

An informal composite phylogenetic tree was adapted from a previous study (Lautenschlager, 2022). Species were added and removed as necessary to be consistent with the dataset of the current study. The final phylogenetic tree was subsequently mapped onto the ternary morphospace using the phytools package^76^ in R 4.3.0.^77^
